# Institutions for the Insane in Prussia, Austria and Germany

**Published:** 1854-09

**Authors:** 


ART. VII.—*Institutions for the Insane in Prussia, Austria and Germany.* By PLINY EARLE, M. D. One of the visiting Physicians to the Lunatic Asylum of the city of New York, late Physician to the Bloomingdale Asylum; Fellow of the College of Physicians and Surgeons of New York, and of the New York Academy of Medicine. New York: Samuel S. & William Wood, 1854. 8vo. pp. 245.

---

The author of this work is favorably known as one of the most enlightened and successful of American practitioners in diseases of the mind. Dr. EARLE has devoted a large portion of his life to the study of this class of affections, and the pages before us are the fruits of travels undertaken for the purpose of extending his knowledge of the treatment of the insane in Europe. As introductory to his account of the institutions for the insane in Germany, he presents a brief sketch of the medical literature of that country on the subject of insanity.

As early as 1783 a magazine chiefly devoted to the doctrines and treatment of mental disorders was commenced, but its existence was ephemeral. In 1805, Dr. REIL established a journal exclusively devoted to those subjects, but receiving but little support from his brethren, it was also soon discontinued. A second periodical by the same editor was not more fortunate. NASSE commenced a journal in 1818, which was published eight years, the influence of which upon the cause of the insane was highly favorable, disseminating as it did a knowledge of their wants, increasing the taste for the study of mental disorders, and awakening in its readers an interest in the improvement of hospitals.

The medical mind of Germany was soon divided between conflicting theories of mental alienation—the *somatic*, the *psycho-somatic*, and the *spiritual*. Dr. EARLE remarks—

"There is not, according to my comprehension of the subject, entire unanimity of sentiment among the advocates of the Somatic doctrine. They all believe that the causative, conditional or necessary lesion in insanity is physical; but while some contend that it must be in the special organ of the mind, the brain, others maintan that it may be in some of the other viscera. Among the supporters of the former theory, is Dr. J. B. Friedreich, of Anspach, in Bavaria, who has published, besides other works, an "Exposition of the Theories upon the Nature and the seat Mental Diseases." He denounces the psychical doctrine of Heinroth as "diabolical." It is not my intention, as this is not the place, even if I were qualified for the undertaking, to enter into a detailed exposition of that doctrine; but having expressed the denunciation of Friedreich, I cannot well avoid a few remarks.

The theory of Dr. Heinroth is based upon the assertion that insanity begins in vice—in a deterioration of the moral sentiments. Now, it appears to me that the great mass of observation furnishes testimony tending to disprove the truth of this assumption. If it be true, why have we not a greater number of insane, since vice, as Dr. Heinroth uses the term, is not generally considered to be of very limited prevalence among mankind? Wherefore are not all criminals affected with mental diseases? How is it that many persons of a blameless character, some of the most noble patterns of purity of life and uprightness of conduct, exemplars in benevolence and piety, are stricken by this awful visitant, while the burglar, the freebooter and the murderer are left unscathed? Whence is it that mental alienation is so common in enlightened nations, and yet so rare among the aborigines? Is it because there is no vice among the American Indians, that, in all their various tribes, as is asserted by authors of undisputed authority, insanity is unknown? If it be so, then let us leave the bright haunts of civilization and hie away to the forests and the prairies: let us crush the foreheads of the Goliaths of Steam and the Printing Press, and shoulder the quiver and bend the bow amid the solitudes of the desert: let us give up the Bible and the Cross and 'bow down to idols of wood and stone.' But, no: no species of sophistical reasoning, how plausible soever upon a superficial examination, no metaphysical hair-splitting, how dexterously soever it may be performed, can ever answer the foregoing propositions in such manner as to reconcile them, in my opinion, with the premises of the argument of Heinroth's theory."

In Germany, as we should expect, the treatment of the insane is conducted upon the most elightened principles. Dr. Earle speaks of the hospitals in high terms of praise—

"In most of the particulars of moral treatment, the German asylums are fully equal to those of the United States. In the most important point of all—if reference be had to curative treatment, or the quietude, order and hygienic condition of the patients—that of manual employment for the inmates, they are superior. The radical source of this superiority lies, undoubtedly, not in the more ardent wishes or the greater efforts of their superintendents for the welfare of the patients—for, in these respects, none can excel the officers of the American asylumns—but in the education of the people, and the nature of the political governments under which they live. Obedience to authority becomes, by education, more a matter of principle or of habit. Furthermore, the asylums are more independent than ours, and the retention and management of patients more optional with the officers."

"In 1830, a period at which there were but five institutions for the insane in the United States, when comparatively little had here been written upon the subject, Dr. Riedel published a description of the Asylum at Prague, in which, so far as regards the moral treatment, he took a position nearly, if not quite, as advanced as that of any author of later days. Dr. Julius, who visited the institutions of Great Britain a few years since, and presented a report thereupon to the Prusian government, expresses his opinion, in the letter already quoted, that, in Germany, "the moral treatment of the insane is more generally understood than anywhere else."

"In regard to the use of restraining apparatus, the opinions and the consequent practice of the several superintendents, are somewhat at variance; yet all, so far as I am acquainted, concur in the belief that those means of restraint should not be wholly abandoned. Drs. Jacobi and Damerow opposes the doctrine of non-restraint promulgated by some of the English physicians; and, upon the same subject, Dr. Roller writes as follows: 'The physicians of Illenau are not unacquainted with that which has been done in England, but they consider the subject as not yet sufficiently investigated to authorize a judgment. They doubt that the many reasons for diminishing the means of restaint, demand also, their entire banishment; and that this, if it be practicable, is advisable.'"

The following extract is interesting:

"A short time before my departure for Europe, I somewhere read the assertion that no blind person was ever known to be insane. Although then in possession of evidence disproving the statement, I nevertheless made it a special point of inquiry at most of the institutions which I visited, and have recorded the results in their proper places. The use of tobacco, among the patients, was another subject of special inquiry; but I found the practice to be so general that the mention of it is frequently omitted. Smoking is invariably permitted, so far as my knowledge extends; snuff is very generally used, to some extent, but I neither saw nor heard of any patient who chewed tobacco."

We have not room to give details of the institutions of Germany devoted to the insane, but we assure our readers, that they will find a rich fund of instruction, and narratives of great interest in these pages of Dr. EARLE.

